# A Neutralizing Human Monoclonal Antibody Protects against Lethal Disease in a New Ferret Model of Acute Nipah Virus Infection

**DOI:** 10.1371/journal.ppat.1000642

**Published:** 2009-10-30

**Authors:** Katharine N. Bossart, Zhongyu Zhu, Deborah Middleton, Jessica Klippel, Gary Crameri, John Bingham, Jennifer A. McEachern, Diane Green, Timothy J. Hancock, Yee-Peng Chan, Andrew C. Hickey, Dimiter S. Dimitrov, Lin-Fa Wang, Christopher C. Broder

**Affiliations:** 1 CSIRO Livestock Industries, Australian Animal Health Laboratory, Geelong, Victoria, Australia; 2 Protein Interactions Group, CCRNP, CCR, NCI-Frederick, NIH, Frederick, Maryland, United States of America; 3 BRP, SAIC-Frederick, Inc., NCI-Frederick, Frederick, Maryland, United States of America; 4 Department of Microbiology and Immunology, Uniformed Services University, Bethesda, Maryland, United States of America; Mount Sinai School of Medicine, United States of America

## Abstract

Nipah virus is a broadly tropic and highly pathogenic zoonotic paramyxovirus in the genus *Henipavirus* whose natural reservoirs are several species of *Pteropus* fruit bats. Nipah virus has repeatedly caused outbreaks over the past decade associated with a severe and often fatal disease in humans and animals. Here, a new ferret model of Nipah virus pathogenesis is described where both respiratory and neurological disease are present in infected animals. Severe disease occurs with viral doses as low as 500 TCID_50_ within 6 to 10 days following infection. The underlying pathology seen in the ferret closely resembles that seen in Nipah virus infected humans, characterized as a widespread multisystemic vasculitis, with virus replicating in highly vascular tissues including lung, spleen and brain, with recoverable virus from a variety of tissues. Using this ferret model a cross-reactive neutralizing human monoclonal antibody, m102.4, targeting the henipavirus G glycoprotein was evaluated *in vivo* as a potential therapeutic agent. All ferrets that received m102.4 ten hours following a high dose oral-nasal Nipah virus challenge were protected from disease while all controls died. This study is the first successful post-exposure passive antibody therapy for Nipah virus using a human monoclonal antibody.

## Author Summary

Nipah virus and Hendra virus are closely related and highly pathogenic zoonoses whose primary natural reservoirs are several species of *Pteropus* fruit bats. Both Nipah and Hendra viruses can cause severe and often fatal disease in a variety of mammalian hosts, including humans. The henipaviruses are categorized as biosafety level 4 (BSL-4) agents, which has limited the development of animal models and the testing of potential therapeutics and vaccine countermeasures. We show here a new ferret model of Nipah virus pathogenesis in which the underlying pathology closely mirrors the illness seen in Nipah virus-infected humans, including both respiratory and neurological disease. We also show that m102.4, a cross-reactive neutralizing human monoclonal antibody that targets the viral attachment glycoprotein, completely protected ferrets from disease when given ten hours after a lethal Nipah virus challenge. This study is the first successful and viable post-exposure passive antibody therapy for Nipah virus using a human monoclonal antibody.

## Introduction

Nipah virus (NiV) together with Hendra virus (HeV) are closely related highly pathogenic zoonoses and are the type species within the paramyxovirus genus *Henipavirus*. Both viruses can cause significant morbidity and mortality in a variety of vertebrate species including humans. The henipaviruses are categorized as zoonotic biosafety level 4 (BSL-4) agents which has limited an extensive examination of their *in vivo* pathogenic features and the development and evaluation of therapeutics or vaccines. NiV and HeV are select agents of biodefense concern that are classified as priority pathogens in category C by the National Institute of Allergy and Infectious Diseases and the Centers for Disease Control and Prevention, with the potential to cause significant morbidity and mortality in humans and major economic and public health impacts (reviewed [1]). Pteropid bats (family *Pteropodidae*), commonly known as flying foxes, are the predominate natural reservoirs for both HeV and NiV (reviewed [2]) although evidence of henipavirus infection has now been reported in a wider range of both frugivorous and insectivorous bats [3],[4].

Since their initial recognition, both viruses have repeatedly re-emerged. In total, 13 HeV outbreaks have occurred in Australia in 1994, 1999, 2004, and 2006–2009, and have always involved horses as an intermediate host with some human infections including four fatalities, the most recent in September 2009 (reviewed [2]) [5],[6]. NiV has also repeatedly caused spill-over events involving hundreds of human cases since 1998 with at least nine recognized occurrences primarily in Bangladesh and India since 2001 (reviewed [2]) with the most recent in March 2008 [7]. Several of the more recent NiV outbreaks have had higher rates of acute respiratory distress syndrome in conjunction with encephalitis, epidemiological findings consistent with multiple rounds of person-to-person transmission [8], higher case fatality rates (∼75%), and direct transmission of virus from flying foxes to humans via contaminated food has been demonstrated [9],[10].

In addition to their highly pathogenic nature, the henipaviruses are also distinguished from all other paramyxoviruses by their unusually broad host tropism. Host cell infection by NiV and HeV requires two membrane-anchored envelope glycoproteins; the attachment (G) glycoprotein which binds the viral receptor, and the fusion (F) glycoprotein which drives virus-host cell membrane merger [11]. The henipavirus G glycoprotein lacks hemagglutinin and neuraminidase activities and the F glycoprotein is a typical class I fusion glycoprotein (reviewed in [12]). The host cell membrane anchored proteins, ephrin-B2 and ephrin-B3 ligands, have been shown to be the receptors employed by the henipaviruses [13],[14],[15],[16]. There are presently no licensed therapeutics available to treat infection caused by the henipaviruses. Recently, we isolated and extensively characterized a neutralizing human monoclonal antibody (hmAb), m102.4, which recognizes the receptor binding domain of the HeV and NiV G glycoproteins. This hmAb potently neutralized both viruses *in vitro* and maintained its biological activity *in vivo* suggesting its possible utility as a passive therapeutic modality following henipavirus infection [17]. Here we report the development and characterization of a novel ferret model of acute NiV infection and associated disease as well as conduct the first henipavirus therapeutic antibody trial using the hmAb m102.4. Together, our data demonstrate that NiV-mediated disease in the ferret closely resembles that seen in humans with the presence of both respiratory and neurological disease. We further demonstrate that m102.4 is an effective post-exposure therapeutic representing the first antiviral drug candidate showing *in vivo* efficacy in treating lethal NiV-mediated disease, and it is the first human mAb therapeutic developed and tested for the treatment of henipavirus infection.

## Results

### Nipah virus infection and disease in ferrets

In humans, disease resulting from NiV infection can vary in intensity from an acute febrile illness or one progressing to severe central nervous and respiratory disease. Pathological findings show systemic vasculitis, necrotizing alveolitis and meningoencephalitis [18],[19]. The disease in experimentally infected cats and hamsters is similar [20],[21]; but in hamsters meninoencephalitis is more prominent, while cats develop an acute respiratory disease [22]. Here, we sought to assess a new ferret model of NiV pathogenesis where our preliminary observations had confirmed susceptibility to NiV infection, with development of systemic vasculitis and involvement of the central nervous and respiratory systems. Ferrets have emerged as a model for several viral respiratory diseases including avian influenza [23], severe acute respiratory syndrome [24]), and morbilliviruses [25], close relatives of henipaviruses [26]. They offer the combined advantages over either of the aforementioned laboratory animal species of being relatively small mammals, while displaying complex behaviors especially in relation to their handlers that may be used to advantage in clinical assessments. They are however also sufficiently large to enable repeated collection of a wide range of clinical samples throughout the course of an experimental infection, as well as administration of potential therapies in a manner similar and consistent with human medicine.

We initiated a NiV minimal infectious dose study (MID_50_) for the purpose of determining an appropriate challenge dose for subsequent work that would reliably productively infect naïve ferrets. Doses of 50, 500, 5,000 or 50,000 TCID_50_ were each administered to groups of two ferrets oral-nasally; the most likely route of natural infection. Based on prior experience with NiV infection in cats [22],[27],[28] and using similar parameters, we defined infection endpoints in ferrets for the purpose of humane euthanasia; these were used as surrogates for lethality. Endpoints were fever plus signs of rapidly progressing clinical illness including both generalized (e.g. inappetance, depression) and localizing (e.g.dyspnea, neurological signs) disease signs. Ferrets that received 50 TCID50 (1–50 and 2–50), and one animal that received 500 TCID50 (3–500) remained well throughout the period of observation, did not shed detectable virus or viral RNA, did not seroconvert, and their tissues were normal at post mortem and histological examination. The remaining ferrets developed fever (4–7 days post-infection (dpi)) and rapidly progressive clinical illness (6–8 dpi). Ferrets inoculated with 50,000 TCID50 (7–50000 and 8–50000) were euthanized 6 and 7 dpi, respectively; those inoculated with 5,000 TCID50 (5–5000 and 6–5000) were euthanized 8 and 10 dpi, respectively, and one ferret inoculated with 500 TCID50 (4–500) was euthanized 9 dpi.

**Figure 1:**
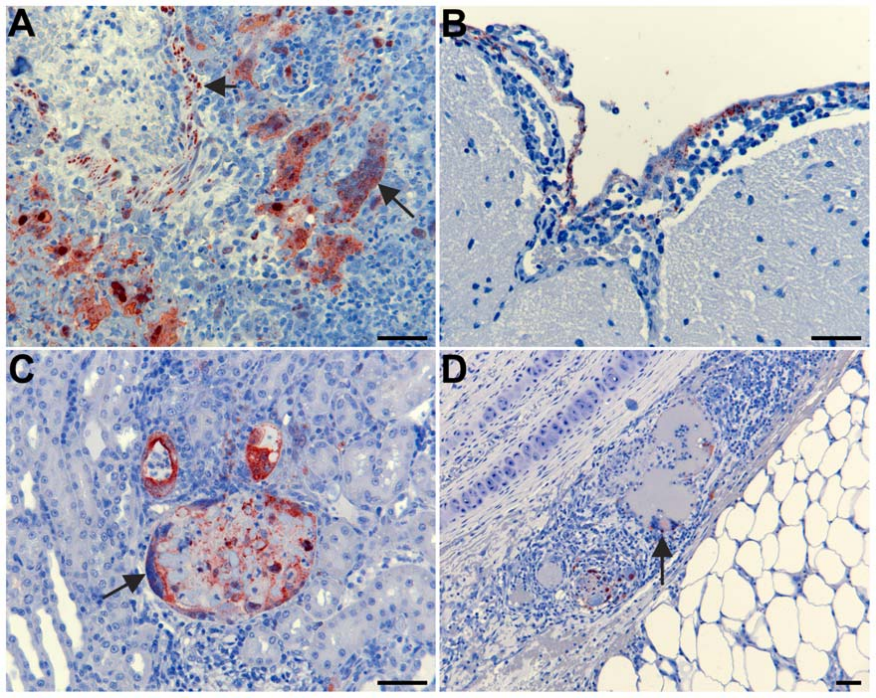
NiV in ferret tissues. Immunohistochemistry was done using a rabbit antiserum against Nipah virus nucleoprotein with haematoxylin counterstain as previously described [22]. (A) ferret 7–50000, lung tissue with severe acute necrotizing alveolitis and vasculitis. Antigen present in syncytia (long arrow) and blood vessel wall (short arrow). (B) ferret 4–500, antigen staining in arachnoid membrane of the meninges, with non-suppurative inflammation. (C) ferret 7–50000, kidney with antigen staining in necrotic glomerular tuft and tubular epithelium; syncytia present in the epithelium of Bowman's capsule (arrow). (D) ferret 4–500, inflammation of the peri-tracheal tissues. Antigen present in blood vessel walls, syncytial cell (arrow). Scale bars = 50 µm.

Clinical signs in affected ferrets included severe depression, cough, serous nasal discharge, dyspnea and subcutaneous edema of the head (8–50000); severe depression, orthopnea, and expiratory dyspnea (7–50000); severe depression, orthopnea and cutaneous ecchymoses (6–5000); severe depression, vomiting and hypothermia (5–5000); and obtunded with tremor and hind limb paresis (4–500). Gross pathological findings comprised varying degrees of subcutaneous hemorrhagic edema of the head and neck, centered upon local lymph nodes in all diseased ferrets; focal raised pin-head hemorrhagic nodules scattered throughout the pulmonary parenchyma; and scattered intra-abdominal petechial hemorrhages. Histopathology of diseased ferrets revealed acute focal necrotizing alveolitis and pulmonary vasculitis, acute glomerular necrosis, focal necrosis of the spleen, and severe diffuse subacute inflammation of the organs and connective tissues of the head and neck. Less commonly, there was mild focal nonsuppurative meningitis (4–500, 5–5000 and 8–50000), focal cystitis (4–500), severe acute necrotizing salpingitis (4–500), acute focal coagulative necrosis of the adrenal cortex (4–500 and 5–5000), and severe acute thyroiditis (6–5000). Two ferrets demonstrated neurological signs (4–500, 5–5000) and had nonsuppurative meningitis. Syncytia were usually present in lesions and often contained abundant viral antigen (**Figure 1**). In mildly affected lymph nodes there was focal mononuclear cell and neutrophilic inflammation of the capsule, accompanied by a zone of subcapsular lymphocyte depletion. In more severely affected lymph nodes, there was severe extensive hemorrhagic and coagulative necrosis, often resulting in the destruction of the entire node with antigen staining mainly in syncytia at the living margin of the necrotic focus (**Figure 1**). Clinically healthy ferrets had no gross pathological changes, and no viral antigen or significant histological lesions were found in any tissues.

### Nipah viral loads and tissue tropism

**Figure 2:**
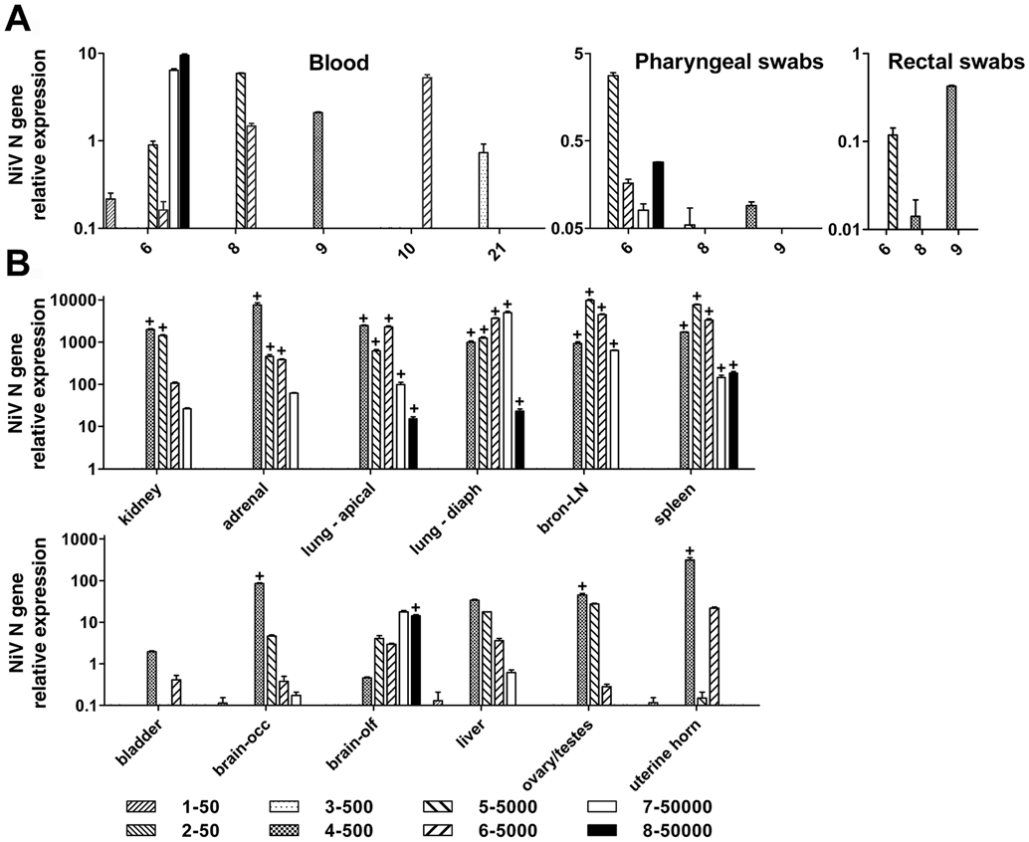
NiV RNA and virus isolation from ferrets post-challenge. Samples were collected in duplicate, RNA was extracted from one replicate and assayed using Taqman PCR detecting NiV nucleoprotein (N) RNA [22]. Samples were assayed in triplicate. Ct values were analyzed against a known NiV cDNA control and results are shown as the average relative NiV N gene expression (Y-axis). Error bars represent the standard deviation. Day of sampling or tissue is indicated (X-axis). Ferret # and NiV dose (TCID_50_) are indicated (inset). (A) Blood, pharyngeal swabs, rectal swabs. (B) Vascular and lymphoid tissues (top panel), other tissues (bottom panel); diaph: diaphragmatic; bron-LN: bronchial-lymph node; occ: occipital; olf: olfactory. For tissue samples with relative NiV N gene expression above 10, virus isolation was performed on duplicate samples. The presence of infectious virus is indicated by the (+) above individual data bars. Virus isolation was not attempted on blood or swabs.

To examine virus dissemination and tissue tropism, NiV N gene RNA was measured in pharyngeal and rectal swabs, blood sampled over the course of infection and in a variety of tissues recovered at necropsy. Among ferrets that developed clinical disease, NiV RNA was detected in the blood (**Figure 2A**) at the time of euthanasia and, in two of these (5–5000, 6–5000), also in the sample collected two days earlier. One ferret (3–500) that had remained healthy during the period of observation demonstrated a low level of viral RNA in blood 21 dpi. Interestingly, this ferret shared a cage with ferret 4–500, from which viral RNA was recovered in pharyngeal and rectal swabs 9 dpi suggesting viral shedding. Viral RNA (albeit some at low level) was detected in pharyngeal swabs from all ferrets demonstrating clinical disease (**Figure 2A**) and in rectal swabs from two of these (**Figure 2A**). Notably, NiV RNA was not detected in any tissues from clinically healthy ferrets. For all other animals with clinical signs, significant levels of viral RNA were detected in adrenal, kidney, lung, bronchial lymph node and spleen tissues (**Figure 2B**). Although at lower relative levels, NiV RNA was also detected in the bladder, liver, ovary or testes, uterine horn and in the brain (**Figure 2B**). All infected ferrets had NiV RNA in the olfactory lobe of the brain and ferret 4–500, which showed tremors and hind limb weakness, had the highest level in the occipital lobe.

Although real-time PCR can help discern virus distribution in an infected animal, it does not discriminate between infectious and non-infectious genetic material. For these reasons, virus isolation was attempted from samples and tissues with NiV RNA relative expression levels above 10. NiV was recovered from almost all tissues that had high levels of NiV RNA (**Figure 2B**, top panel) as indicated by the (+) above individual data bars. Lungs and lymphoid tissues were sites of extensive virus replication. Virus was also isolated from tissues with lower NiV RNA levels, including the occipital lobe, uterine horn and ovary from ferret 4–500 and the olfactory lobe from ferret 8–50000 (**Figure 2B**, bottom panel). For ferrets where NiV was isolated from brain, histological lesions included nonsuppurative meningitis.

### Antibody m102.4 prevents NiV disease in ferrets

**Figure 3:**
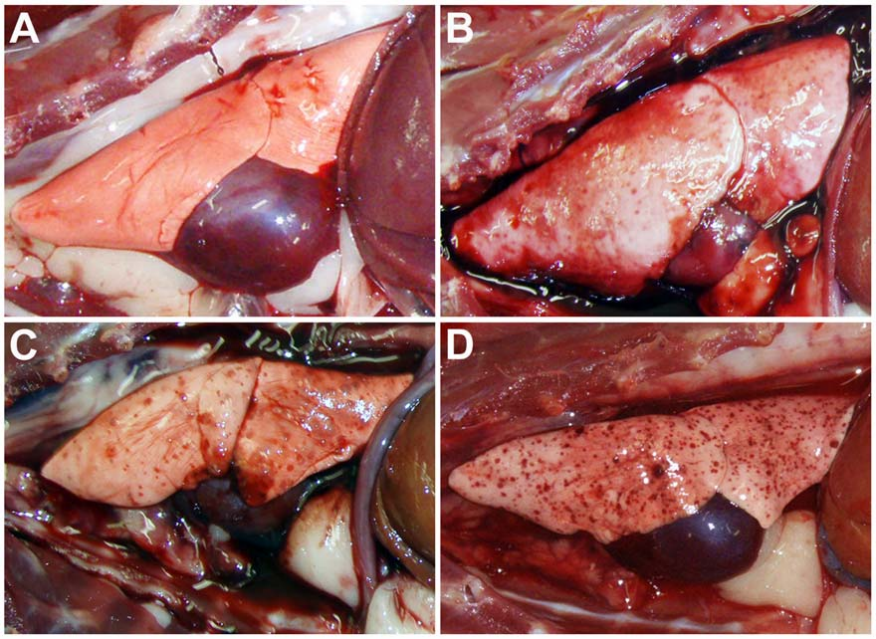
Gross pathology of the lung post-mortem in ferrets treated with m102.4 prior to NiV challenge. (A) Ferret 21-pre; 20 days post infection (dpi): no signs of illness, disease or lesions: healthy ferret lungs. (B) Ferret 23-pre; 13 dpi: delayed disease onset, scattered small pinpoint lesions. (C) Ferret 24-pre; 13 dpi: delayed disease onset, scattered small pinpoint lesions. (D) NiV-infected control ferret; 8 dpi: typical disease onset, extensive pinpoint lesions.

**Figure 4:**
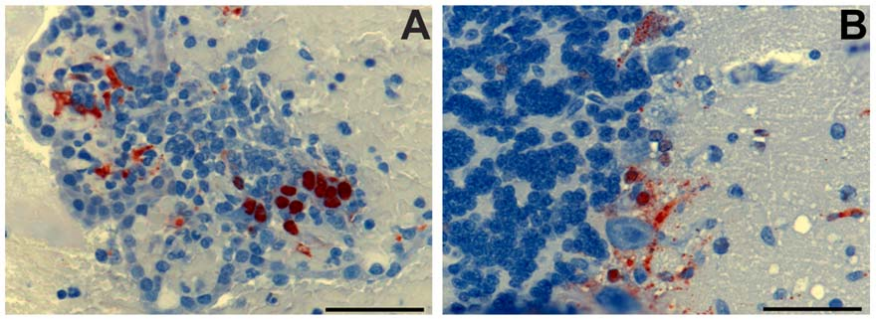
Immunohistochemistry demonstrating NiV antigen in brain tissue in ferrets treated with m102.4 prior to NiV challenge. (A) Ferret 23-pre; 13 dpi: viral antigen in ependymal epithelium of the lateral ventricle, associated with focal hemorrhage. (B) Ferret 24-pre; 13 dpi: single focus of viral antigen in the cerebellum. Scale bar = 50 µm.

Recently, we described a hmAb, m102.4 which engages the receptor-binding site of the viral attachment G glycoprotein, potently neutralizes HeV and NiV *in vitro*, and retained its biological activity *in vivo* [17]. For the evaluation of m102.4 efficacy in the ferret model two time points were selected, 24 hrs before (pre-) or 10 hrs after (post-) NiV challenge. Each treatment group contained three ferrets except for the control group (n = 2). At the indicated time, 50 mg of m102.4 was administered via intravenous catheter. One ferret in the post-challenge group (28-post) was given m102.4 intraperitoneally due to difficulty in venous catheterization. Control ferrets (22-con and 25-con) were given PBS. Ferrets were challenged oral-nasally with a 5,000 TCID_50_ dose of NiV (ten-fold the MID_50_ as determined by the dose-ranging study above). By 6 dpi, ferrets 22-con, 25-con and 23-pre were febrile (>40°C) and control animals started to demonstrate signs of clinical illness similar to those seen in the initial MID_50_ study. By 8 dpi the controls were severely depressed, had subcutaneous edema of the head and cutaneous hemorrhages and they were euthanized. By 8 dpi, 24-pre and all ferrets in the post-group were also febrile with variable levels of depression and suppression of play activity; a notable sign of abnormal ferret behavior. At 10 dpi, the temperatures of 23-pre and 27-post had started to fall, and each demonstrated moderate (23-pre) or mild (27-post) edema of the throat. 24-pre and 26-post remained febrile with no localizing signs. By 13 dpi, ferrets 23-pre and 24-pre were depressed and inappetant with cutaneous ecchymoses; ferret 23-pre had marked hind limb paresis and generalized tremor and both animals were euthanized. All other ferrets (three in the post- and one in the pre-group) were well and free of any disease signs and remained so until the end of the study (**Table 1**). Upon study completion (20 dpi) all surviving animals were euthanized. For the controls and two ferrets in the pre-challenge group that succumbed to infection, gross and microscopic pathology revealed findings similar to those described above in the MID_50_ study. However, in ferrets 23-pre and 24-pre the frequency of pinpoint hemorrhagic lesions observed in the pulmonary parenchyma was reduced and lesions were much smaller suggesting the disease progression in the respiratory tract had been dampened (**Figure 3**), consistent with their survival to 13 dpi. Histopathological findings in controls and in the two diseased pre-challenge group ferrets were similar to those described in the MID_50_ study. However, for ferret 23-pre, NiV antigen was present in ependymal tissue (**Figure 4A**) and for ferret 24-pre NiV antigen was present within neurons and neuropil of the cerebellum (**Figure 4B**), further supporting the suggestion that ferrets could be an appropriate model for NiV-mediated meningoencephalitic disease. No significant pathological abnormalities were found in any of the surviving ferrets.

**Table 1:** Clinical scoring and outcome of NiV-infected ferrets from m102.4 efficacy trial.

DPI	Ferret	Fever	Depression	Play Activity	Grooming	Inappetance	Additional symptoms	Total weight loss
6	22-con	+	+	x	x	−		
	25-con	+	+	x	x	+		
	23-pre	+	−	-	-	−		
8	**22-con**	+	+	xxx	x	−	**-diarrhea**	**8.7%**
	**25-con**	+	+	xxx	x	+	**-blood in mouth**	**12.3%**
	23-pre	+	+/−	x	x	+/−	-shivering	
	24-pre	+	−	-	-	−		
	21-pre	−	−	-	-	−		
	26-post	+	−	x/-	-	−		
	27-post	+	+/−	x	-	−	-diarrhea	
	28-post	+	+/−	x	-	−		
10	23-pre	+	+/−	x/-	x	+	-sneezing, weeping eye, diarrhea	
	24-pre	+	+	x	-	+	-nasal discharge	
	21-pre	−	−	-	-	−		
	26-post	+	−	x/-	-	−		
	27-post	−	+	-	-	−	-sneezing	
	28-post	−	−	-	-	−		
13	**23-pre**	+	+	x/-	-	+	**-diarrhea, crusting nasal discharge, tremors, hind limb paralysis**	**16.6%**
	**24-pre**	+	+	xxx	-	+	**-diarrhea, crusting nasal discharge**	**4.7%**
	21-pre	−	−	-	-	−		
	26-post	−	−	-	-	−		
	27-post	−	−	-	-	−		
	28-post	−	−	-	-	−		

DPI: days post infection; (−) no symptom; (+) present; (x) presence of abnormality (x: moderate; xx: substantial; xxx: severe). Bold text indicates animals that required euthanasia due to NiV-mediated disease. For ferret 28-post, m102.4 was administered intraperitoneally.

### Nipah viral loads and tissue tropism in m102.4 treated ferrets

**Figure 5:**
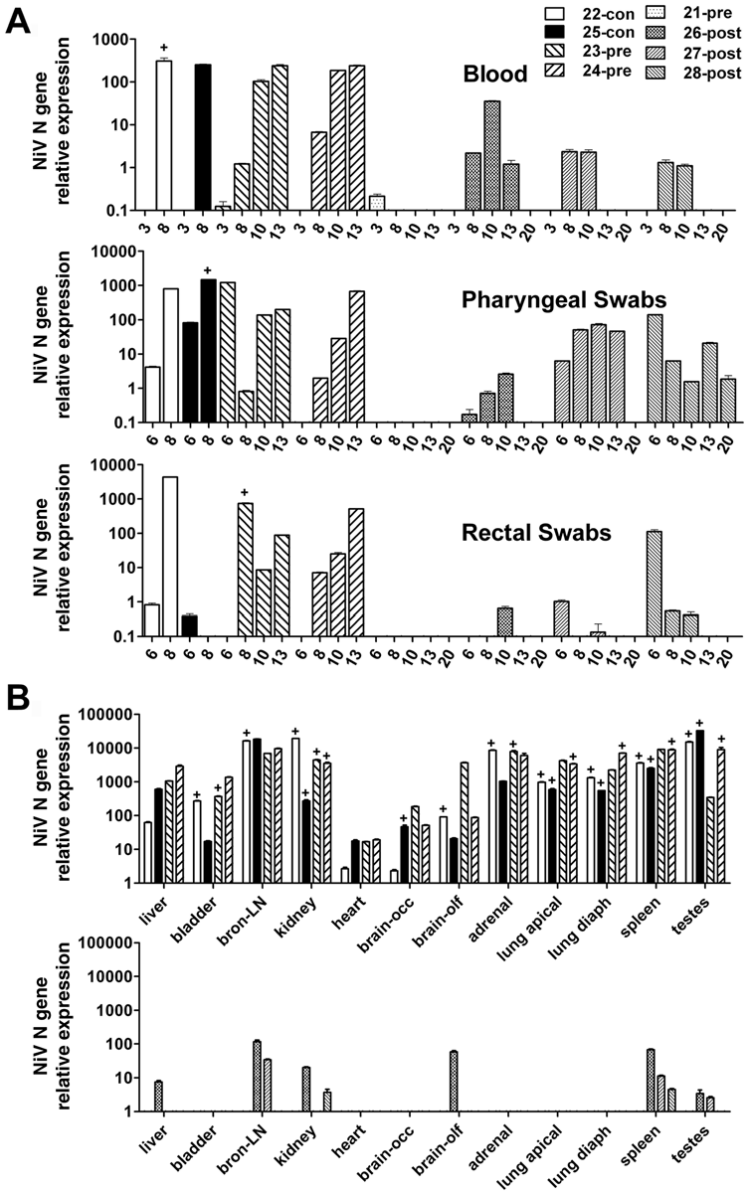
Viral loads in ferrets from m102.4 efficacy trial. All samples were collected in duplicate. RNA was extracted from one replicate and assayed using Taqman PCR assay detecting NiV nucleoprotein (N) RNA. RNA samples were assayed in triplicate. The Ct values were analyzed against a NiV cDNA control and results are shown as the average relative NiV N gene expression (Y-axis). Error bars represent the standard deviation. Day of sampling or tissue sample is indicated on the X-axis. Ferret # and treatment group (control (con), pre- or post-challenge) are indicated (inset). (A) Blood, pharyngeal swabs, rectal swabs. (B) Tissues from diseased ferrets (top panel) or healthy ferrets (bottom panel); diaph: diaphragmatic; bron-LN: bronchial-lymph node; occ: occipital; olf: olfactory. Virus isolation was attempted from duplicate samples where relative NiV N gene expression levels were above 10. The presence of infectious virus is indicated by the (+) above individual data bars.

Blood, swabs and tissues were tested for the presence of NiV RNA and results are shown in **Figure 5**. Ferret 21-pre, the only animal to survive in the pre-challenge group, had detectable levels of viral RNA in the blood 3 dpi (**Figure 5A**) whereas all other samples were negative. All other ferrets had significant viremia by 8 dpi with the highest levels of NiV RNA in the controls. By 13 dpi, viral RNA levels in ferrets 23-pre and 24-pre were similar to those found in controls whereas NiV RNA levels had decreased in ferret 26-post and were undetectable in the other post-challenge group ferrets. Viral RNA levels in oral swabs were highest among the controls and pre-challenge group; however, post-challenge group ferrets shed virus over the course of infection (**Figure 5A**). NiV RNA levels in rectal swabs were low except in 23-pre and 24-pre (**Figure 5A**). Together these data demonstrated that all treated ferrets (except 21-pre) had significant amounts of systemic and mucosal NiV for at least 10 dpi. Controls and the two diseased pre-group ferrets had similar high levels of viral RNA in all tissues (**Figure 5B**). Conversely, 21-pre and all post-challenge group ferrets had significantly reduced levels of viral RNA (**Figure 5B**). Virus isolation was attempted on all samples with detectable NiV RNA (**Figure 5**). On day 8 post-infection, NiV was isolated from the blood of one control animal (22-con), from a pharyngeal swab from the other control animal (25-con) and from a rectal swab from ferret 23-pre. All other blood and swab samples were negative for virus isolation despite the high levels of viral RNA detected. Virus was isolated from the majority of control animal tissues and the two diseased pre-group ferret tissues; whereas, for 21-pre and all post-group ferrets, tissues were negative for NiV. Together, these data demonstrate that treatment with m102.4 reduced viral replication and spread, and provided a significant therapeutic benefit leading to the survival of all infected animals in the post-challenge group.

### *In vivo* m102.4 concentration

**Figure 6:**
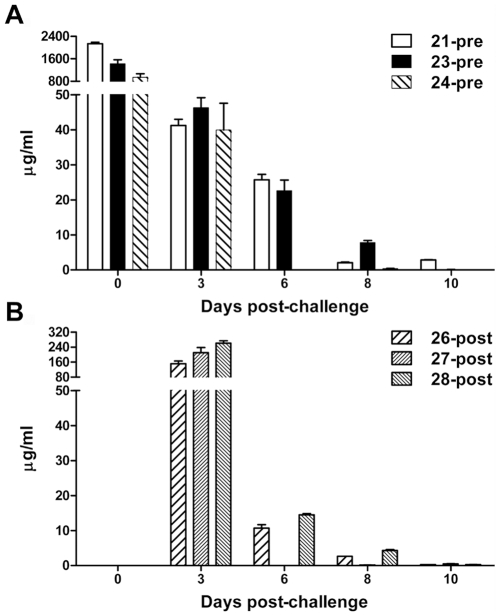
*In vivo* m102.4 concentrations in ferrets following NiV challenge. Blood was collected at various time points post-challenge (X-axis). Serum was collected and frozen at −80°C until the completion of the study. All sera were γ-irradiated to facilitate removal from the BSL4 laboratory. Sera was diluted and assayed using a NiV G-specific microsphere assay described previously [17],[37]. An m102.4 standard curve ranging from 500 ng/ml to 0.5 ng/ml and all sera samples were assayed in duplicate simultaneously. Serum m102.4 concentrations were extrapolated from the standard curve using non-linear regression analysis and results are shown as the average concentration. Error bars represent the standard deviation. Ferret # and treatment group (pre- or post-challenge) are indicated (inset). (A) m102.4 administered 24 hrs pre-challenge. (B) m102.4 administered ten hours after post-challenge.

**Figure 7:**
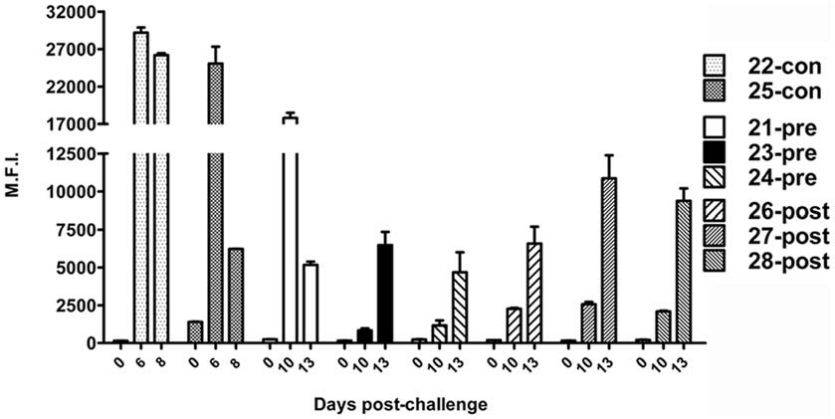
Ferret immune responses to NiV G. Blood was collected at various time points post-challenge, as indicated by the X-axis. Serum was collected and frozen at −80°C until the completion of the study. All sera were γ-irradiated to facilitate removal from the BSL-4 laboratory. Ferret sera were diluted and assayed using the NiV G-specific microsphere assay described previously [37]. Ferret # and corresponding treatment group (control (con), pre- or post-challenge) are indicated in the legend. Median fluorescence intensities (M.F.I.) are shown on the Y-axis. All assays were performed in duplicate and the mean M.F.I. is shown. Error bars represent the range of M.F.I. for each sample.

To evaluate whether a correlation between m102.4 concentration and protection existed, levels of m102.4 in serum were measured (**Figure 6**). Coincidently, ferret 21-pre had the highest levels of serum m102.4 pre-challenge along with the longest-lasting serum levels (**Figure 6A**) and was the only animal from this group that survived. By 3 dpi, all pre-challenge group ferrets had significantly lower m102.4 concentrations compared to the post-challenge group (**Figure 6B**). Importantly, m102.4 concentrations on day 3 correlated with survival and may indicate the existence of a critical therapeutic window following virus exposure. By 6 dpi, m102.4 levels in all animals had dropped and were the lowest among the post-challenge group. Interestingly, on 6 and 8 dpi, 23-pre had higher serum m102.4 levels than those in the post-group and succumbed to disease (13 dpi) whereas 27-post had low levels of m102.4 on these days and survived infection. Antibody specific for NiV G was measured in serum post-challenge. NiV-G specific antibodies were detected in controls by 6 dpi (**Figure 7**). Ferret 21-pre had high levels of G-specific antibodies 10 dpi which likely reflected m102.4. For all other ferrets, NiV G-specific antibodies were low on 10 dpi with higher amounts of antibody present in the post-challenge group. By 13 dpi, NiV-G specific antibody responses had increased further in all ferrets with the highest levels found in the 27-post and 28-post ferrets.

### Monoclonal antibody resistance and virus strain specificity

In the present study, we have described m102.4, a potent neutralizing human monoclonal antibody. Its mechanism of action involves binding a single epitope within the receptor binding domain of the henipavirus G glycoprotein and affectively blocking receptor engagement and virus infection. HeV and NiV cause acute and often fatal disease in animals and humans and although virus neutralization escape mutants are unlikely in 7–10 days; we evaluated whether resistant virus was present in ferrets 23-pre and 24-pre. Virus isolation was repeated on all tissues homogenates in the presence and absence of m102.4. Virus was isolated from all samples in the absence of m102.4 as before; however, no virus was isolated from tissues when m102.4 was present (data not shown). Together, these data demonstrate that the virus circulating in ferrets 23-pre and 24-pre at the time of euthanasia was still highly sensitive to m102.4 and no evidence of escape mutants was found. In addition, we also examined m102.4 neutralizing activity against different isolates of NiV and HeV, including, NiV-Malaysia, NiV-Bangladesh, HeV-1994 and HeV-Redlands. Importantly, m102.4 neutralized all viruses potently: HeV-Redlands was completely neutralized at 1.25 µg/ml m102.4 and all other isolates were even more sensitive with complete neutralization occurring at 0.63 µg/ml m102.4. These data support the potential broad applicability of m102.4 as a post-exposure therapeutic option for henipavirus-infected individuals.

## Discussion

A new ferret model of NiV infection and pathogenesis has been developed in the present study. Unlike the cat and hamster models, the ferret model is unique and exhibits both severe respiratory and neurological disease, and generalized vasculitis. NiV-mediated disease in humans has been described as a systemic endothelial infection accompanied by vasculitis, thrombosis, ischaemia and necrosis [18]. Histopathological changes in infected individuals were especially noted in the central nervous system (CNS) with widespread presence of viral antigen in neurons and other parenchymal cells in necrotic foci in the CNS and also in endothelial cells of affected blood vessels. Both vasculitis and endothelial infection was also seen in most organs examined. In ferrets, clinical disease included vascular fibrinoid necrosis in multiple organs, necrotizing alveolitis, syncytia of endothelium and alveolar epithelium, and necrotizing lymphadenitis. Histopathological lesions included severe focal necrotizing alveolitis, vasculitis, degeneration of glomerular tufts, and focal necrosis in a wide-range of other tissues. Significant quantities of viral antigen in blood vessel walls and syncytial cells were frequently present. Viral antigen was present in neurons and infectious NiV was isolated from multiple organs including the brain. Several animals also demonstrated significant neurological disease. A widespread presence of NiV antigens in neurons and other parenchymal cells in the CNS was not as extensive as in NiV-infected ferrets as compared to infected humans, and this could relate to the length of disease course between these experimental versus natural infections. We speculate that a more widespread infection in the CNS would have developed if animals were allowed to succumb to disease without euthanasia. Overall, NiV-mediated disease in ferrets has all the hallmarks of NiV-infected humans and represents a novel relevant animal model.

Recent vaccine studies have demonstrated that henipavirus G-specific antibodies are critical for protection from disease [22],[28]. Evidence of passive protection against NiV, and more recently HeV, challenge using hamster polyclonal antiserum or murine mAbs reactive to NiV glycoproteins was provided in the hamster [29],[30],[31]. However, in those prior studies, both the challenge virus and antibodies were administered by intraperitoneal injection either simultaneously or immediately before or following challenge. Here we examined a neutralizing fully-human mAb, with the potential for human use, in a consistently susceptible animal model where challenge and drug delivery mimicked a potential real-life scenario.

The hmAb m102.4 is an ideal therapeutic, targeting a critical functional domain of the G glycoprotein, and is a fully human IgG. We achieved full protection from NiV-mediated disease in ferrets who received a single infusion of antibody 10 hrs post-challenge and protection in one animal treated prior to challenge. Ferret 21-pre was the only survivor in the pre-group and it had the highest pre-challenge level of m102.4 and the best antibody longevity. Uniquely, fever, clinical signs and virus were not detected at any time in this animal, with the exception of an extremely low level viremia 3 dpi. These findings suggest that the combination of an initial high level of antibody and subsequent longevity may provide protection. For the remaining ferrets, higher levels of m102.4 on day 3 post-challenge correlated with protection from disease whereas levels thereafter did not, potentially highlighting the first 3 days following infection as an important therapeutic window. On day 3, lower m102.4 concentrations in the pre-group may not have been sufficient to neutralize all virus, allowing the initiation of infection. In the post-group the higher concentrations 3 dpi may have sufficiently neutralized enough virus and/or prevented widespread dissemination of virus, providing a window for the animal to mount an effective immune response. In the post-challenge group, the host immune system may have had an opportunity (10 hrs) to initiate a primary response before antibody-mediated depletion of virus. In the pre-challenge group, the antibody-virus complexes most likely formed almost immediately following challenge and the primary or innate response may have been dampened. As m102.4 levels decreased over time and virus replication initiated, the post-challenge group may have had a more potent secondary response allowing survival. Indeed, the animals in the pre-challenge group developed severe disease 7 days after m102.4 levels decreased, a timeline similar to the disease progression in naive animals. Whether primary or secondary, the antibody responses in the post-challenge group appeared earlier (10 dpi) and were more robust.

The survival of all ferrets in the post-challenge group represents a major step forward towards a viable passive antiviral therapeutic for treating henipavirus infection. Since m102.4 targets the receptor-binding domain of G [32] and is a conformation-dependent antibody, we believe it will maintain efficacy *in vivo* during the course of its application in an infected host. Given the acute nature of henipavirus infection and its receptor specificity, mutation and escape from m102.4 sensitivity seems unlikely. Mutations generating m102.4 neutralization escape variant viruses would likely also acquire defects in receptor binding and fitness. Our data support this notion as no escape mutants were found in treated animals that succumbed to disease. Further examination of m102.4 has confirmed its potent cross-reactive neutralization activity against the NiV-Malaysia [33], the original HeV-1994 [34], the recent HeV-Redlands [5] and NiV-Bangladesh isolates [35]. Examination of m102.4 effectiveness in treating animals exposed to these henipavirus isolates will be important. In future studies, it will also be critical to increase the number of animals studied and further explore the therapeutic window post-challenge. Although 10 hrs is relevant to a known exposure, such as a laboratory accident, it will be important to define the limits of the therapeutic window. These data represent the first human antibody therapy successfully evaluated *in vivo* for prevention of lethal henipavirus infection and strongly suggests m102.4 may prove to be a useful therapeutic used to treat disease in people caused by these important emerging pathogens.

## Materials and Methods

### Animal handling and husbandry

Eight adult (1–2 year old) ferrets were used for the NiV minimal infectious dose study and eight adult ferrets were used for the *in vivo* m102.4 efficacy study. All animal studies were endorsed by the CSIRO, AAHL, Animal Ethics Committee. Ferrets were housed in pairs in cages in a BSL4 room, fed twice daily with a complete premium dry food and provided with water *ad lib*. For implantation of temperature transponders, NiV challenge, administration of m102.4 and specimen collection, ferrets were anaesthetised by intramuscular injection of ketamine (Ketamil, Troy Laboratories, New South Wales (NSW), AU) and medetomidine (Domitor, Pfizer Animal Health, NSW, AU) at dose rates of approximately 3 mg/kg and 0.03 mg/kg, respectively. For anesthetic reversal, atimepazole (Antisedan; Novartis, Pendle Hill, NSW, AU) was given intramuscularly at 50% the dose of medetomidine. Following challenge, ferrets were assessed daily and scored out of 10 for a range of clinical observations, including alertness, playfulness, curiosity, depression, food consumption, feces production and respiration rate. Real-time monitoring of body temperature for all animals was done using transponders implanted subcutaneously [28]. Rectal temperatures and body weights were recorded on sampling days. Once ferrets were clinically assessed to be exhibiting signs of severe disease from which natural recovery was considered unlikely, animals were euthanized by intravenous injection of sodium pentabarbitone.

### Virus isolate and challenge

Ferrets were inoculated oronasally with a low passage NiV isolate (NiV-Malaysia; EUKK 19817; stock virus titer 4.3×10^6^ TCID_50_/ml) [36]. For the MID_50_ study NiV doses were: 5×10^4^, 5×10^3^, 5×10^2^ and 50 TCID_50_ (2 ferrets per dose). Based on the outcome of the MID50 study, for the m102.4 efficacy study all ferrets were challenged with 5×10^3^ TCID_50_.

### Preparation and administration of m102.4

The m102.4 hmAb was prepared as previously described [17]. Ferrets were anaesthetised and a 20-g intravenous catheter placed in the left jugular vein. m102.4 was administered via catheter by slow infusion over 4 minutes to 6 ferrets (50 mg per ferret); 3 ferrets received antibody 24 hours before NiV challenge and 3 ferrets received antibody 10 hours after challenge. Two ferrets received PBS, one at 24 hours before challenge and the other at 10 hours after challenge. Catheters were withdrawn and animals were allowed to recover from the anaesthesia.

### Sample collection post-challenge

Blood, oral swabs and rectal swabs were collected 6, 8, 10, 13 and 20 or 21 days post-infection (dpi) for the purpose of assisting in establishing the infection status of each animal. For the m102.4 study an additional sample was collected during incubation period (3 dpi). Two aliquots of whole blood were removed from each sample and serum was collected and aliquoted. One aliquot of whole blood was added to RiboPure lysis buffer (Ambion Inc. Austin, TX, USA) containing sodium acetate. Duplicate swabs were placed in AVL viral lysis buffer (Qiagen Pty Ltd, Clifton Hill, Victoria, AU) or PBS. After euthanasia, tissue samples were collected aseptically from lung (apical and diaphragmatic lobes), brain (olfactory and occipital lobes), heart, bronchial lymph nodes, spleen, liver, kidney, bladder, and adrenal gland. In females, the uterine horn and ovaries were collected and in males the testes were collected. Tissues were either fixed in 10% neutral buffered formalin, submerged in RLT lysis buffer (Qiagen Pty Ltd) containing β-mercaptethanol and 1 mm stainless steel beads (BioSpec Products Inc., Bartlesville, OK, USA) or submerged in PBS containing 1 mm stainless steel beads. For the MID_50_ study, all specimens were placed at −80°C. For the m102.4 efficacy study, all blood, swab and tissue samples were processed immediately for RNA extraction and duplicate samples in PBS were stored at −80°C for virus isolation.

### RNA isolation

RNA was purified from blood cells using the RiboPure-Blood kit (Ambion Inc.), from swabs using the QIAamp viral RNA kit (Qiagen Pty Ltd) and from tissues using the RNeasy Mini kit (Qiagen Pty Ltd). Prior to processing, tissues were homogenized for 2 cycles of 30 sec using a Mini-Bead Beater (Biospec Products Inc.) and centrifuged to pellet debris.

### NiV Taqman PCR assay

Taqman PCR assays were preformed as previously described [28]. Samples were amplified in a GeneAmp 7500 Sequence Detection System (Applied Biosystems, Foster City, CA). Ct values representing NiV nucleocapsid (N) gene expression were analyzed and data were recorded as relative NiV N gene expression levels.

### Virus isolation

Virus isolation was performed as previously described [28] and only attempted from specimens positive for NiV RNA.

### Histopathology and immunohistochemistry

Tissues were processed by routine histological methods and sections of tissue were stained with hematoxylin and eosin to examine histopathological changes. Separate sections were stained by immunohistochemical techniques as previously described [20] using a rabbit polyclonal antiserum against NiV nucleoprotein.

### Measurement of m102.4 and NiV G-specific antibodies in ferret serum

Multiplexed microsphere assays were performed as previously described [37]. A Luminex® 100 IS™ machine and MiraiBio software (MiraiBio Group, South San Francisco, CA) were utilized for all assays: Master Plex CT v1.0 for data acquisition and MasterPlex QT v 2.0 for data analysis. All samples were assayed simultaneously and concentrations were extrapolated from a standard curve using non-linear regression analysis (GraphPad Software, San Diego, CA).
